# Systemisers *are* better at maths

**DOI:** 10.1038/s41598-018-30013-8

**Published:** 2018-08-02

**Authors:** Paola Bressan

**Affiliations:** Dipartimento di Psicologia Generale, Università di Padova, Via Venezia 8, 35131 Padova, Italy

## Abstract

People with superior mathematical abilities turn out to have an autism spectrum disorder more often than others do. The empathising-systemising theory proposes that this link is mediated by these individuals’ stronger tendency to systemise (detect patterns, derive rules), along with the fact that mathematics is the perfect example of a rule-based, lawful system. This account, however, requires that individuals from the general population who are more inclined to systemise be better at maths than those who are less inclined to do so. Based on the scant available evidence, this has been argued not to be the case. The data presented here show, for the first time, that systemising tendencies do predict both self-assessed maths skills (201 participants) and mathematical intelligence (151 participants), before and after controlling for nonmathematical intelligence, sex, and occupation (social sciences vs biological/physical fields). These findings support the empathising-systemising theory and the “hyper-systemising” explanation of autism.

## Introduction

If people do not believe that mathematics is simple, it is only because they do not realize how complicated *life* is.

—John von Neumann,

*First national meeting of the Association for Computing Machinery*, *1947*^1^

Our brain has evolved to make sense of what is happening and predict what will happen next. According to a popular theory^2^, this process relies on two hardwired specialised skills, systemising and empathising. The systemising module deals with the physical, inanimate world and equips the brain to understand its rules. The empathising module comes prepackaged with the special abilities needed to understand the social world—mainly other people’s behaviour, a highly complex and erratic system—and react to it.

The systemising and empathising mechanisms are naturally set at different levels in each of us^3^, and this has a number of consequences. For example, the systemising mechanism might be set so high that the individual is unable to cope with any exception to rules and becomes distressed by change. This “hyper-systemising” configuration has been argued to underlie the behavioural symptoms of people with autism spectrum disorders^3,4^. As expected of hyper-systemisers, individuals with autism spectrum disorders loathe chaos of any description and display restricted interests—focusing on only a few aspects of the physical, and eschewing the social, environment^5^. Even when systemising and empathising are in the normal range, however, some people may be more strongly drawn to systemising and others to empathising. In particular, starting from a very young age, females tend on average to empathise more and males to systemise more^6^.

Systemising means observing how events relate to one another, detecting patterns, and deriving rules. Thus, systemisers will be attracted by systems that are lawful and predictable (usually inanimate: machines, tools, timetables) and will be frustrated by systems that are less so, like other people’s behaviour.

A system that is perfectly lawful is mathematics, and a drive to systemise has been suggested to foster talent in it (e.g.^2^). There is some indirect evidence for a connection between the choice of mathematics as a study area and what is purportedly the extreme expression of systemising, autism. Relative to nonmathematicians, mathematicians score higher on the autism questionnaire^7^ and are three- to sevenfold more likely to receive, or to have a family member that has received, a diagnosis of autism spectrum condition^8^.

Yet, remarkably, there is currently no evidence for a relation between systemising tendencies and mathematical skill. This link appears to have been directly tested twice, with disappointing results in both cases. In a sample of 93 female university students, systemising scores were associated with higher self-reported confidence in the ability to solve maths problems, but not with better performance in an actual maths test^9^. Systemising scores were unrelated to mathematical achievement—under the form of either calculation or problem solving skills—in a sample of 112 children too^10^. These null results have been regarded as highlighting some serious problem with both the empathising-systemising theory and the hyper-systemising explanation of autism^9^.

Here I present new data showing that the systemising score predicts both self-assessed mathematical skills and mathematical intelligence, defined as the ability to solve mathematical problems under time pressure. I argue that these findings support the empathising-systemising theory and the hyper-systemising explanation of autism.

## Methods

### Participants

Of the 201 individuals that participated in the study, 99 were men and 102 women (mean and median age 23.6 and 23 years, range 18–60 years). Most of them were university students of disciplines requiring different levels of systemising—mainly psychology and engineering. Participants were recruited and tested individually. The experimental protocol was approved by the Psychological Research Ethics Committee of the University of Padua and was in accordance with the relevant guidelines and regulations. Informed consent was obtained from all participants.

### Materials and procedure

#### Mathematical intelligence

Two different measures of participants’ mathematical skills were obtained, one subjective and one objective. The first was a self-assessment of mathematical ability (“how good are you at maths, on a 0 to 10 scale?”). The second was the score in the Italian version of the arithmetic subtest of the Wechsler Adult Intelligence Scale Revised (WAIS-R). This requires solving, under time pressure, arithmetic problems from easy (e.g., “What is the total of 4 plus 5 apples?”) to relatively hard (e.g., “If 8 machines can finish a job in 6 days, how many machines are needed to finish it in half a day?”).

#### Nonmathematical intelligence

General, nonmathematical intelligence was assessed with the Italian version of the similarities subtest of the WAIS-R. This involves solving, under time pressure, non-mathematical problems from easy (“In what way are an orange and a banana alike?”) to relatively hard (“In what way are praise and punishment alike?”).

#### Systemising tendencies

Systemising tendencies were measured with the Italian version of the 25-item version of the Systemising Quotient (SQ-Short^11^). The questionnaire assesses an individual’s drive and preference for systemising across a range of domains; it contains items such as “I am fascinated by how machines work” and “I find it difficult to understand information the bank sends me on different investment and saving systems” (reverse-coded). Responses are given on a 4-point Likert scale (definitely agree, slightly agree, slightly disagree, and definitely disagree). Whereas the original systemising quotient scoring assigned zero to all responses going in a direction opposite to systemising, I preserved the full four-point scores to retain all information and avoid reducing scale reliability (see^12^ for a similar argument). Each “definitely” systemising response scored 2 points and each “slightly” systemising response scored 1; each “definitely” anti-systemising response scored −2 points and each “slightly” anti-systemising response scored −1. The systemising score was the sum of all points and could therefore range from −50 to 50.

#### Procedure

Data were collected as part of a larger study. The tasks of interest here were presented in the following order: self-assessment of mathematical ability, WAIS-R arithmetic, WAIS-R similarities, and systemising quotient questionnaire. Of the 201 individuals that participated in the study, 151 completed all the tasks above whereas 50 were presented with all of them except the WAIS-R tests. Participants were individually tested in the same laboratory.

### Data coding and analysis

Individuals who study or work in fields that require interest for rule-based systems (such as Science, Technology, Engineering, and Mathematics: STEM disciplines) should have a higher systemising tendency than those who do not. These fields also tend to require stronger mathematical proficiency, and/or help to build it. In principle, then, a relationship between systemising and maths ability might simply emerge as a side-effect of systemisers being drawn to these disciplines. For this reason, participants’ occupation (area of study or work) was also considered in the analyses.

Occupations were ordered by increasing degree of systemising required, as follows: humanities, social sciences (including psychology, economics, and management), biological sciences (including medicine, biology, natural sciences, and environmental sciences), physical-system fields (including engineering, technology, mathematics, physics, geology, and chemistry). Psychology was placed among the social rather than biological sciences because the overwhelming majority of psychology students at the University of Padua aspire to become psychotherapists or clinical psychologists.

Of the 169 participants whose occupation could be classified, about 2% were in the humanities, 44% in the social sciences, 15% in the biological sciences, and 39% in physical-system fields. Because of its very small numerosity (4 participants: 2 students of law and 2 students of linguistic and cultural mediation), the humanities category was collapsed with the social sciences category. (Order effects, such as Spearman correlations, remained basically the same if the humanities group was retained as a separate category or discarded altogether.) The percentage of women in the social-, biological-, and physical-system categories was respectively 59%, 44%, and 43%.

For bivariate correlations, occupation was entered as an ordinal variable (three levels: social, biological, and physical careers). For linear multiple regressions, that do not admit ordinal predictors, occupation was entered as a categorical variable by collapsing the biological- and physical-sciences groups. This subdivision (two levels: social vs biological/physical careers) split participants roughly in half.

The relationships between the systemising score and all other measures were analysed with bivariate correlations or Student’s *t*. The relationships of interest were further explored with multiple regressions that used the systemising score to predict each of the two mathematical skills measures (self-assessment and WAIS-R) while controlling for nonmathematical intelligence, occupation, and sex. Because some of the independent variables were correlated, I checked for potential collinearity issues but found none.

All quoted probabilities are two-tailed and rounded to one significant digit^13^ (i.e., the first non-zero digit after the decimal point).

### Data availability

The data that support the findings of this study are publicly available at figshare.com/s/db9e7b1e38578c245a2c.

## Results

**Figure 1:**
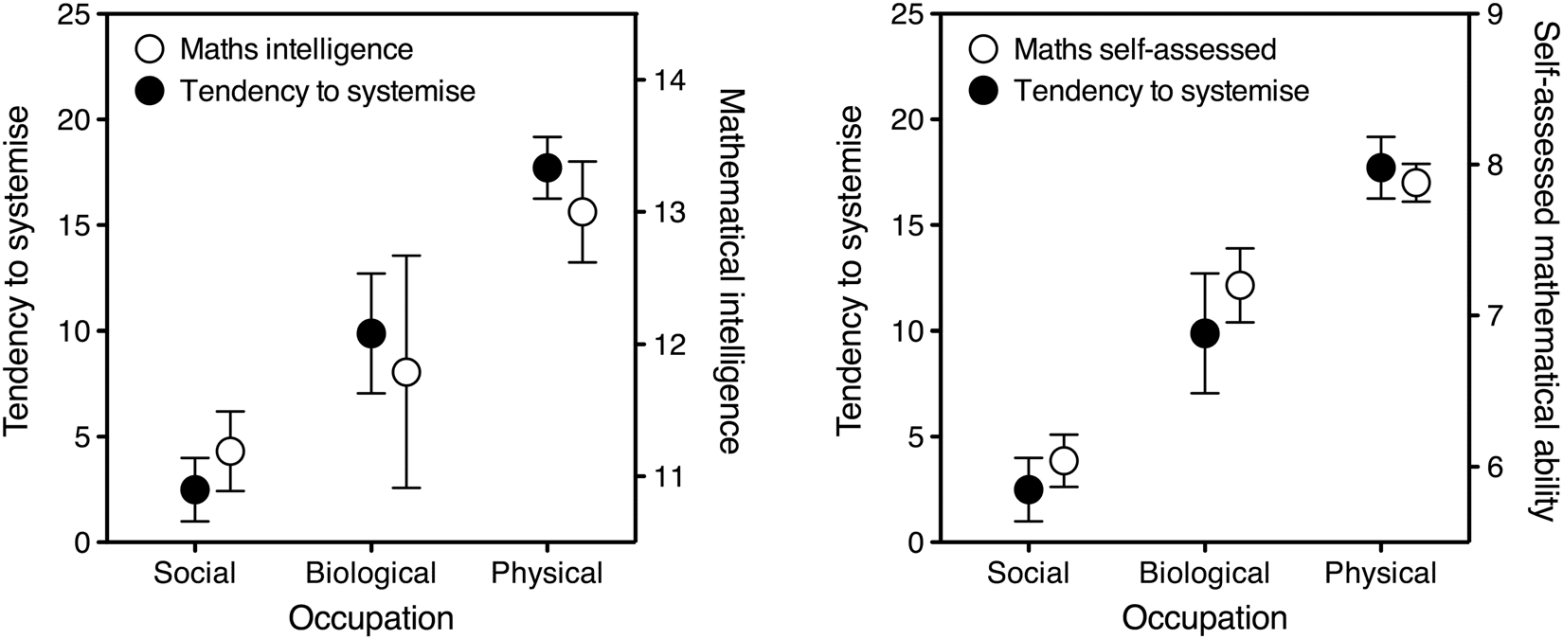
Tendency to systemise, mathematical intelligence (left panel) and self-assessed mathematical ability (right panel) as a function of occupation field (social, biological, physical). Systemising scores for participants whose occupation could be classified (*N* = 169) are plotted on the left Y axis, mathematical-skill data are plotted on the right Y axis. The relationship between left and right Y-axis scaling is arbitrary. Error bars indicate one standard error of the mean. To prevent overlap, open symbols have been nudged horizontally. Left panel: Mathematical intelligence is expressed as the score in the arithmetic subtest of the Wechsler Adult Intelligence Scale Revised (WAIS-R). The large standard error for the biological-sciences occupation is due to the small number of participants in this group that took the WAIS-R test (*N* = 14). Right panel: Self-assessed mathematical ability is expressed as participants’ answer to the question “how good are you at maths, on a 0 to 10 scale?”.

Systemising was predicted by sex, with men scoring on average higher than women (13.2 vs 6.3, *t*_199_ = 3.5, *p* = 0.0005). Systemising was predicted by occupation too (Fig. 1), with increasing mean scores for social-, biological-, and physical-career participants (2.5 vs 9.9 vs 17.7, Spearman *rho* = 0.49, *N* = 169, *p* < 0.0001).

### Systemising and mathematical intelligence

Systemising correlated significantly with the WAIS-R arithmetic score (*r* = 0.31, *N* = 151, *p* = 0.0001).

The WAIS-R arithmetic score also depended on sex, being higher for men than for women (12.1 vs 11.1, *t*_149_ = 2.4, *p* = 0.02), and on occupation, being higher for participants in biological/physical than in social fields (12.7 vs 11.2, *t*_126_ = 3.0, *p* = 0.003, even though the choice of collapsing biological and physical careers was conservative: see left panel of Fig. 1 for a decompressed representation). A multiple regression was used to test whether systemising provided a unique contribution to mathematical intelligence after adjusting for sex and occupation.

Although sex and occupation predicted WAIS-R arithmetic both separately and in combination, neither did when systemising was added to the regression model (*R* = 0.36, *F*_3,124_ = 6.3, *p* = 0.0005; systemising, *β* = 0.25, *p* = 0.01; occupation, *β* = 0.12, *p* = 0.2; sex, *β* = 0.10, *p* = 0.3).

### Systemising and self-assessed mathematical ability

Systemising correlated significantly with self-assessed mathematical ability (Pearson *r* = 0.39, *N* = 201, *p* < 0.0001; Spearman *rho* = 0.41, *N* = 201, *p* < 0.0001).

The combination of systemising, sex, and occupation (social vs biological/physical fields) predicted 30% of the variance in self-assessed mathematical ability (*R* = 0.55, *F*_3,165_ = 23.8, *p* < 0.0001), but only systemising (*β* = 0.17, *p* = 0.03) and occupation (*β* = 0.46, *p* < 0.0001) contributed to the overall effect, whereas sex did not (*β* = 0.006, *p* = 0.9). Indeed, results were virtually identical if only systemising and occupation were entered as predictors. If occupation rather than sex was dropped from the regression model, the combination of systemising and sex predicted 15% of the variance (*R* = 0.39, *p* < 0.0001), with only systemising (*β* = 0.39, *p* < 0.0001) and not sex (*β* = 0.002, *p* = 1) contributing to the effect.

**Figure 2:**
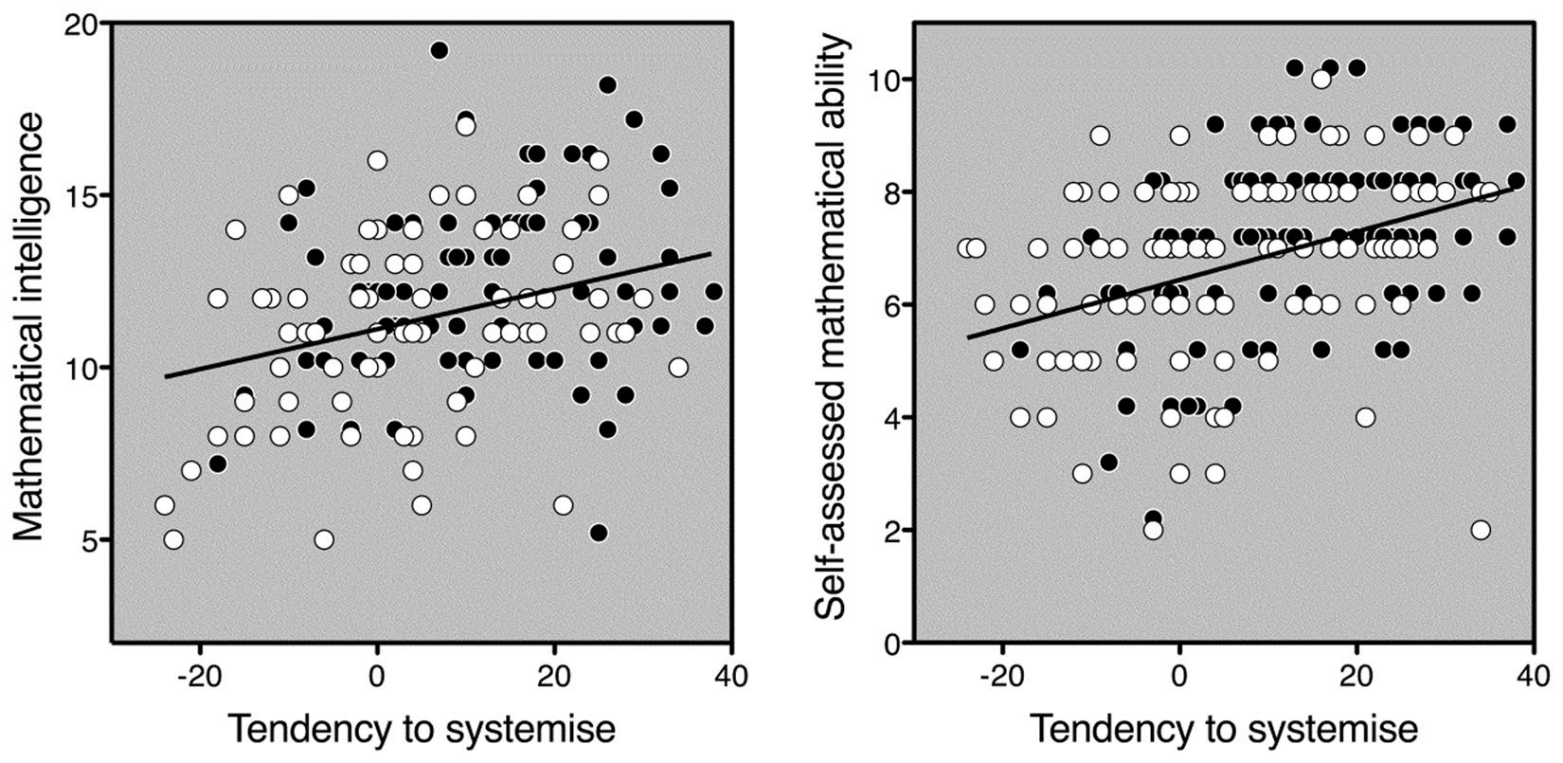
Mathematical intelligence (left panel) and self-assessed mathematical ability (right panel) as a function of the tendency to systemise. Data are plotted separately for women (white symbols) and men (black symbols). Black symbols have been nudged vertically to diminish overlap and ensure visibility of all data points. The regression line is a fit to all data points regardless of participant’s sex (i.e., regardless of symbol colour).

None of the regressions changed in any meaningful way when nonmathematical intelligence (in the form of WAIS-R similarities scores) was added as a predictor. In fact, nonmathematical intelligence turned out to be unrelated not only to systemising (*r* = −0.06, *N* = 151, *p* = 0.5), but also to mathematical intelligence (*r* = 0.07, *N* = 151, *p* = 0.4) and self-assessed mathematical ability (*r* = −0.02, *N* = 151, *p* = 0.8). The correlations between systemising and either mathematical intelligence or self-assessed mathematical ability were not significantly different for men and women, both |*z*|_s_ ≤ 1.02, both *p*_s_ > 0.3. Figure 2 illustrates the relationship between tendency to systemise and mathematical-skill measures, separately for each sex.

## Discussion

In a sample of students of a diverse range of disciplines, the systemising score predicted both self-assessed mathematical skills (201 participants) and mathematical intelligence (151 participants). This remained true after controlling for nonmathematical intelligence, for occupation (social vs biological/physical careers), and for sex.

Notice that, although in the WAIS-R arithmetic test men scored higher than women (confirming previous reports^14^), this gap disappeared when differences in the tendency to systemise were controlled for. This implies that, on average, men are better at maths than women because, on average, men are more driven to systemise than women. To put it another way, the qualities that are associated with maths skills are better expressed by the systemising questionnaire than by biological sex.

Symmetrically, participants whose interests lay in physical systems (mostly engineers) did better than those whose interests lay in social sciences (mostly psychologists) in the WAIS-R arithmetic test. Yet the effect of being an engineer or a psychologist on maths intelligence became nonsignificant when individual differences in the tendency to systemise were controlled for.

The tendency to systemise did in fact depend strongly on occupation. Students in the physical sciences have been reported to score higher than students in the humanities in either the 75-item^15^ or the 40-item^16^ systemising quotient questionnaires. The study presented here, that compared engineers to psychologists (whose curriculum does contain some math, at least in the form of statistics) rather than to humanities students, represents a more conservative test of the same point. Note that being good at dealing with the rules of the nonhuman, natural environment and being good at maths are arguably the main reason why engineers and physical scientists were attracted to their fields to start with. Thus, controlling for occupation when testing the correlation between systemising and maths skills is a highly—possibly overly—conservative choice. Still, whereas self-assessed mathematical ability remained firmly linked to the occupation domain, actual mathematical intelligence did not.

The finding that systemisers are better at maths is quite different from the null results recently obtained with children^10^. That study used both calculation and problem-solving measures as indices of mathematical achievement and found no correlation of either with children’s systemising scores. Yet no significant sex difference emerged for any of these indices, in disagreement with well-established data (for calculation and problem solving, cf^14^; for systemising, cf^17^)—potentially suggesting lack of power. (Incidentally, calculation skills are actually better, rather than worse, in girls than in boys, a gap that disappears in adolescents and adults^14^; no correlation with systemising scores should therefore have been expected for them.)

The other study^9^ that explored the relationship between systemising and maths performance, finding none, was done on female psychology students entering an introductory statistics course. However, the maths performance test did not reflect the ability to solve problems but rather the knowledge of maths basics deemed essential to succeed in a statistics course, such as addition, division, exponentiation, fractions, simple equations and such^18^. Familiarity with these specific notions may derive more from previous schooling, assisted by one’s general intelligence and diligence, than from the strength of one’s systemising drive. Indeed, in the same study^9^, participants’ self-reported level of confidence in solving maths problems did rise with the systemising score. Thus that study, if anything, supports the credibility of the empathising-systemising theory rather than shattering it.

The existence of a relationship between systemising and mathematical intelligence has an important bearing on theory. It has been argued that the association between autism spectrum disorders and better maths skills^7,8^ could be driven by a stronger tendency to systemise^2^. This line of thought, however, requires that ordinary people who tend to systemise more are better at maths. The data presented here show, for the first time, that this is indeed the case.

From a practical point of view, these findings endorse the notion that we may be able to help children learn—and perhaps even like—mathematics if we encourage, through games and specific activities, the development of their pleasure to systemise.
